# SnapShot: Embryo models

**DOI:** 10.1016/j.stemcr.2021.04.012

**Published:** 2021-05-11

**Authors:** Nicolas Rivron, Jianping Fu

**Affiliations:** 1 Institute of Molecular Biotechnology of the Austrian Academy of Sciences (IMBA), Vienna BioCenter (VBC), Vienna, Austria; 2 Department of Mechanical Engineering, Department of Biomedical Engineering, and Department of Cell & Developmental Biology, University of Michigan, Ann Arbor, MI 48198, USA

Embryo models derived from cultured stem cells represent a scientific, biomedical, and ethical opportunity. With distinct levels of completeness and capabilities to model different developmental stages, embryo models offer an experimental platform for *in vitro* research into the principles of early embryogenesis of mice, humans, and other mammalian species.

To view this SnapShot, open or download the PDF.

